# Procedures of data merging in precision cancer medicine: the PRIME-ROSE project

**DOI:** 10.2340/1651-226X.2026.44889

**Published:** 2026-01-06

**Authors:** Henk van der Pol, Tina Kringelbach, Maria Martin Agudo, Gabriel Bratseeth Stav, Gro Live Fagereng, Marta Fiocco, Ragnhild Sørum Falk, Victoria Homer, Soemeya Haj Mohammad, Hans Timmer, Loic Verlingue, Åslaug Helland, Kristoffer Rohrberg, Ulrik Lassen, Sarah Halford, Katriina Jalkanen, Tanja Juslin, Matthew G. Krebs, Julio Oliveira, Edita Baltruškevičienė, Kristiina Ojamaa, Kjetil Taskén, Hans Gelderblom

**Affiliations:** a Department of Medical Oncology, Leiden University Medical Centre, Leiden, The Netherlands; b Mathematical Institute, Leiden University, Leiden, The Netherlands; c Department of Oncology, Copenhagen University Hospital – Rigshospitalet, Copenhagen, Denmark; d Institute for Cancer Research and Division of Cancer Medicine, Oslo University Hospital, Oslo, Norway; e Princess Maxima Center, Utrecht, The Netherlands; f Department of Biomedical Data Science, Leiden University Medical Center, Leiden, The Netherlands; g Oslo Centre for Biostatistics and Epidemiology, Oslo University Hospital, Oslo, Norway; h Cancer Research (UK) Clinical Trials Unit, University of Birmingham, Birmingham, United Kingdom; i Centre Léon Bérard, Lyon, France; j Institute of Clinical Medicine, University of Oslo, Oslo, Norway; k Department of Clinical Medicine, University of Copenhagen, Copenhagen, Denmark; l Cancer Research UK, London, United Kingdom; m Helsinki University Hospital Comprehensive Cancer Center, Helsinki, Finland; n Division of Cancer Sciences, The University of Manchester and The Christie NHS Foundation Trust, Manchester, United Kingdom; o Portuguese Oncology Institute of Porto, Porto, Portugal; p National Cancer Insitute, Vilnius, Lithuania; q Tartu University Hospital, Tartu, Estonia

**Keywords:** Data sharing, DRUP-like clinical trials (DLCTs), European project, Precision Cancer Medicine (PCM), inclusion rate

## Abstract

**Background and purpose:**

As more interventional clinical trials in Precision Cancer Medicine (PCM) are introduced, molecular descriptions of tumours have led to multiple subtypes, even within common tumour types. Therefore, the main limitation of these trials is the small number of eligible patients to assess the clinical benefit. The PRIME-ROSE project addresses this limitation by pooling data from multiple European Drug Rediscovery Protocol (DRUP)-like clinical trials, such that slowly accruing cohorts are accelerated. To achieve this task, a well-documented commonly approved procedure for data merging needs to be established.

**Patient/material and methods:**

Data sharing is achievable when there is an organisation that includes people from different disciplines who can navigate institutional and country-specific information and governance requirements. Furthermore, alignment of all the study procedures are needed before data are shared. Next, the process of merging data requires harmonisation and standardisation. Implementation of the Observational Medical Outcomes Partnership (OMOP) Common Data Model (CDM) facilitates future data aggregation.

**Results:**

By aggregating data from European DRUP-like clinical trials, cohorts are completed that were unable to do so in stand-alone studies. Since initiation, the PRIME-ROSE project monitors over 300 cohorts across more than 20 treatments encompassing over 1,000 patients. At least 20 cohorts have progressed after interim analysis.

**Interpretation:**

Data sharing across European trials is feasible and enhances the advancements of PCM studies. The methodologies developed in the PRIME-ROSE project provide a foundation for future data integration efforts in PCM clinical trials, underscoring the viability of conducting robust trials in a global context.

## Introduction

Numerous Precision Cancer Medicine (PCM) trials have been conducted globally for several years, including the TAPUR trial in the United States, the BELIEVE trial in Japan, and the Drug Rediscovery Protocol (DRUP) in the Netherlands [1–3]. In Europe, several national DRUP-Like Clinical Trials (DLCTs) have been launched [4, 5], and these countries are now collaborating within the PRIME-ROSE project (Precision Cancer Medicine Repurposing System Using Pragmatic Clinical Trials), funded by the European Commission [5, 6]. The DLCT network is now pooling patient data to accelerate the assessment of drug efficacy in cohorts defined by specific treatment, tumour type and biomarker combinations.

The PRIME-ROSE project and other initiatives in Europe advance PCM trials by creating a platform to share and analyse data [7]. By pooling patient information from the individual trials, the likelihood of completing cohorts increases significantly. The project continues the work of PCM4EU (Personalised Cancer Medicine for all EU Citizens), which initiated the implementation of DLCTs across Europe as part of Europe’s Beating Cancer Plan. Furthermore, as new treatments are developed, it is crucial to have a platform that allows rapid assessment of efficacy across different tumour types and biomarkers, using standardised methods and guidelines to support future data sharing.

In this report, we focus on the procedures for data merging in PCM and share the knowledge gained in the area of data pooling. Results show that data integration can be done effectively, and the first cohorts are currently under analysis.

## Patients/material and methods

The aim of this report is to document the data merging process between the DLCTs. Specifically, we will address patient safety considerations, data alignment, and standardisation. In addition, we will discuss the process of data sharing and the rules established for merging cohorts. All trials have signed a Data Sharing Agreement (DSA), and share data in one central server, which facilitates the merging process.

### Organisation

**Figure 1:**
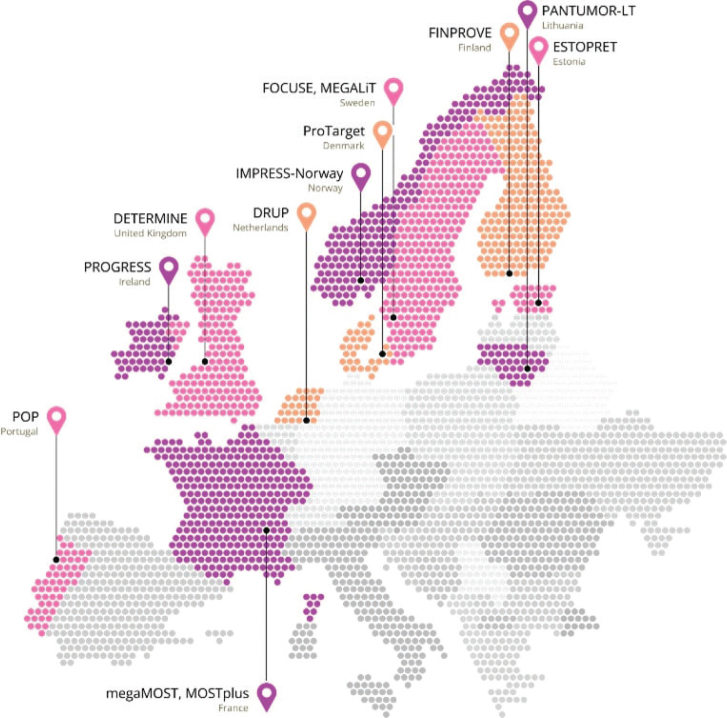
An overview of all ongoing, ended and soon-to-start DRUP-like clinical trials in Europe. DRUP in the Netherlands., ProTarget in Denmark. FINPROVE in Finland. IMPRESS-Norway in Norway. FOCUSE and MEGALiT in Sweden., MOSTplus and megaMOST in France. DETERMINE in the United Kingdom. PROGRESS in Ireland.POP in Portugal. ESTOPRET in Estonia. PANTUMOUR-LT in Lithuania.

Currently, there are 11 active or soon-to-start DLCTs across Europe (see Figure 1). The network is steadily expanding by sharing documents and expertise. To further support the development of new trials, a buddy system has been implemented in the PCM4EU project [5], providing mentoring and guidance to emerging studies. The governance structure, detailed in the Data Management Plan (DMP), ensures that each trial retains ownership of its data, while the data processor serves as a central data hub. Inclusion numbers from each trial are shared monthly to track the inclusion rate in the merged cohorts. The complete dataset for each patient in a cohort, defined within the Data Sharing Protocol (DSP), is shared with the central processor upon completion after approval by each national Principal Investigator (PI). Moreover, each DLCT also has the option to withhold data for specific cohorts. A publication committee, which includes the PI from each data sharing trial, grants approval to proceed with the analysis and publication of shared cohorts. The DLCT governance structure is designed to respect and integrate each participating trial’s existing national governance frameworks.

### Monthly meetings

Key representatives from each trial meet monthly to review patient recruitment status and assess completed cohorts across DLCTs. Before each meeting, updated inclusion rate specific data from each trial’s electronic Case Report Forms (eCRFs) is sent to the data processor, who then standardises, harmonises, and merges the data. This monthly data is limited to support ascertainment of recruitment status within cohorts. These meetings provide an overview of cohort recruitment, enables cross-trial decisions to either advance or close cohorts based on efficacy data from each trial. Furthermore, the monthly meetings facilitate discussions to align tumour types and biomarker definitions for specific cohorts, and assign responsibility for final analysis and reporting of closed cohorts.

### Publication plan

An updated overview of merged cohorts is available to all members of the DLCT community through a secured website. Additionally, the conclusion from each meeting is shared monthly in the internal newsletter along with decisions regarding the continuation of cohorts, closing of cohorts, and potential opening of expansion or new cohorts.

A final report evaluating the treatment effect, based on the statistical analysis plan (SAP), will be published after a cohort is closed to accepting new patients, either through completion or early closure. This will be done in agreement with each trial that provided data, as outlined in the DMP, and in coordination with the (pharmaceutical) company providing the drug.

### Alignment of each DLCT

The prerequisite for merging data between DLCTs, and clinical trials in general, is the alignment of key components of each protocol to ensure that the analysis is carried out with homogenous data, thus minimising potential biases. This section discusses alignment of endpoints, schedule of activities, inclusion- and exclusion criteria, and adverse events (AEs).

### Endpoints

Although the complete list of primary and secondary endpoints varies between the DLCTs (Table 1), the trials share five primary and secondary endpoints:

**Table 1:** An overview of the most shared endpoints between the trials.

Endpoints	DRUP	ProTarget	IMPRESS	POP	FOCUSE	FINPROVE	PANTUMOUR-LT	ESTOPRET	DETERMINE	MOST
Treatment-related grade ≥ 3 and SAEs	**X**	**X**	**X**	**X**	**X**	**X**	**X**	**X**	**X**	**X**
Progression-Free Survival	**X**	**X**	**X**	**X**	**X**	**X**	**X**	**X**	**X**	**X**
Overall Survival	**X**	**X**	**X**	**X**	**X**	**X**	**X**	**X**	**X**	**X**
DCR (objective complete response, partial response or stable disease) at 16 weeks of treatment initiation according to established response criteria	**X**	**X**	**X**	**X**	**X**	**X**	**X**	**X**	**X** ^*^	**X** ^**^
Duration of time on drug	**X**	**X**	**X**	**X**	**X**	**X**	**X**	**X**	**X**	**X**
Percentage of patients that are included and treated based on their molecular tumour profile	**X**	**X**	**X**	**X**	**X**	**X**	**X**			
Description of mutational concordance between first (pre-treatment) biopsy and subsequent biopsies in the study	**X**	**X**	**X**	**X**	**X**			**X**		

(*) The DETERMINE trial observes DCR at 24 weeks. (**) The MOST trial observes DCR at 9 and 18 weeks.

1. Treatment-related grade greater than or equal to 3 and Serious Adverse Events (SAEs)
2. Progression-Free Survival (PFS)
3. Overall Survival (OS)
4. Disease Control Rate (DCR) assessed as complete response (CR), partial response (PR), or stable disease (SD) at 16 weeks of treatment initiation according to established response criteria.
5. Duration of time on drug.

Regarding endpoint 4, while all DLCTs measure DCR, the MOST trial observes disease control at 9 and 18 weeks, and the DETERMINE trial at 24 weeks post treatment initiation. For DETERMINE, disease control information is also measured at 16 weeks (for arms where the treatment is given on a 4-weekly cycle) or at 18 weeks (for arms where the treatment is given on a 3-weekly cycle). Therefore, treatment information is shared from these timepoints to allow for better harmonisation of endpoints. Additionally, Time on Treatment/Drug, and the percentage of patients treated according to their molecular profile are endpoints common to 80% of the DLCTs (see Table 1 for an overview of the most shared endpoints).

All trials participating in the PRIME-ROSE project will collect Health-Related Quality of Life (HRQoL) data using standardised instruments (e.g. EORTC QLQ-C30, EQ-5D, Risk Attitude Questionnaire and WISP QoL surveys). The assessment is collected by most trials in the screening phase, every 3 months during and at the end of treatment. This provides sufficient data for analysis (Table 2).

**Table 2:** The DLCTs share similar study procedures.

Study Procedures	1–28 days	W8	W16	Every 3 months	EOT
AE/SAE assessment	**+++**	**+++**	**++**	**+++**	**++**
Tumour assessment	**+++**	**+++**	**++**	**+++**	**+**
Laboratory assessments	**+++**	**+**	**+**	**++**	
Physical examination	**+++**			**+**	**+**
Concomitant medication	**+++**			**+**	**++**
HRQoL	**+**			**+**	**+**
Informed consent	**+++**				
Medical history	**+++**				
ECG	**+++**				
Pregnancy test	**+++**				

Key: W = week, EOT = End of Trial, (+++) = shared by all trials, (++) = shared by 10 out of 11 trials, (+) = shared by eight or nine out of 11 trials. First column: screening phase. Second column: Treatment phase. Third column: End of Treatment phase.

### Inclusion and exclusion criteria

To assess merged cohorts, it is essential to ensure that each DLCT has a comparable pool of patients eligible for inclusion. Most trials have general and drug-specific inclusion and exclusion criteria. A complete overview of the general inclusion and exclusion criteria in all trials has been conducted and shared within the consortium. Differences in the general criteria must be considered when merging cohorts. The most significant differences are as follows:

- The MOST trial includes only patients with ECOG performance status of 0–1, and the DETERMINE trial includes only patients with ECOG status 0–1 (those with a performance status of 2 are considered on an individual basis). This is in contrast to an ECOG performance status of 0–2 inclusion criteria in other trials. This may result in a higher proportion of healthier patients, which may affect the results.
- Some trials require a mandatory fresh frozen biopsy prior to starting treatment. This could influence patient populations, as some patients might be excluded based on this criterion.
- Variation in health-related requirements between trials. Although considered low risk, as all trials require that patients are likely to benefit from treatment. An evaluation of medical history and concomitant therapy should still be performed.

If a cohort is completed and analysed, the difference in criteria needs to be considered. This depends on the patient population of each cohort.

## Adverse events

When combining data from different trials, a common AE dictionary is essential. AEs are defined in all protocols as any undesirable experience that a patient experiences during the study, regardless of whether it is related to the study treatment or not. The safety reporting and handling of AEs and SAEs are consistent across all trials.

In addition, Suspected Unexpected Serious Adverse Reactions (SUSARs) are managed identically. Grading AEs severity is performed using either the National Cancer Institutes (NCI) Common Terminology Criteria for Adverse Events (CTCAE) version 4.03 or version 5.0, with only the DRUP trial using version 4.03. A complete overview of all differences in AEs is shared between the consortium.

### Schedule of activities

The DLCTs are aligned with the original DRUP protocol [3], and the schedule of activities is similar across trials, facilitating data merging. Table 2 shows the common core set of activities for all trials, which is sufficient to cover the variables for merging and evaluating endpoints, except for HRQoL.

### Merging data

#### Harmonising and standardisation of the data

Each DLCT uses its local eCRF system to capture and store patient data, ensuring the completeness and accuracy of patient information. The selected variables, as described in the DSP, must be harmonised and standardised. The DSP contains a list of variables that are shared at the end of a patient’s participation in their respective trial, monthly basis, and upon cohort completion. Note, some of these transfers may be combined.

To securely manage data in such a way that it is safe and complies with privacy regulations, the Services for Sensitive Data (TSD) platform is used. This ensures that data collection, storage, analysis, and sharing comply with GDPR Articles 6 and 9. Researchers who have been assigned the privileges can access the data from each trial through the TSD. The TSD project administrator for PRIME-ROSE provides a periodic import link to the data stewards of each DLCT, allowing them to upload data into the TSD. In this step, the data stewards of the individual DLCTs collects full records of patient level data before anonymisation to ensure data quality. The data processor pre-processes the data and harmonises the individual outcome variable to ensure consistency across all DLCTs.

Harmonisation is required to ensure that the variable lists from each DLCT are consistent. We define *harmonisation* as the process of ensuring that data formats across DLCTs are uniform (e.g. converting different clinical outcome data formats to a standard format). The data from each trial is collected in a structured manner through the eCRF system, and the standard variables are uniformly measured and recorded, for example, tumour assessment through RECIST. *Standardisation* involves aligning data according to a central or established standardisation method (e.g. AE grades are measured by the CTCAE).

Data collected in the eCRF systems are structured data. However, harmonisation of tumour type and biomarker definition remains one of the main challenges. To address this, we have developed a standardised list of biomarkers and tumour types, with definitions agreed upon by all DLCTs. More specifically, the biomarker definition will be harmonised according to a predefined list of actionable targets and assessed by a molecular biologist from each trial during cohort evaluation.

#### Standardisation to a Common Data Model

The combined data will be standardised using a Common Data Model (CDM) to facilitate future data analysis and data sharing outside the trial network. In this project, we will standardise using the Observational Medical Outcomes Partnership (OMOP) CDM [8]. The OMOP CDM is an open-source CDM by the Observational Health Data Sciences and Informatics (OHDSI) community. Each outcome variable is first harmonised to an OMOP standard. Once all outcome variables have been harmonised and standardised, merging data from DLCTs, data quality checks and statistical analyses can be done efficiently through existing tools developed for OMOP. Moreover, standardising to the OMOP CDM enables federated data merging with similar trials.

#### Merging data for monitoring inclusion

Once the variables from different trials are aligned and standardised, cohorts with the same tumour type, biomarker target, and treatment can be merged. This allows for pooling of results across the PRIME-ROSE project. The cohort merging is approved manually during the monthly meetings to ensure accurate merging. Inclusion numbers for each cohort are updated monthly and presented at the evaluation meetings.

### Decision making process

When merging the data from different DLCTs, there are three critical decision triggering time-points:

1. The first decision involves choosing the cohorts to merge from the individual trials. Due to the high complexity of particularly genomic biomarkers and cancer type definitions, this is not limited to the biomarker/tumour type/treatment-definition of a cohort. For example, cohorts may be tumour-agnostic, histology-specific or tumour types that are naturally grouped together. Therefore, the decision to merge cohorts is on a cohort-by-cohort basis and is documented and decided during the monthly meetings.
2. Cohorts that are monitored in the monthly meetings may undergo interim assessments to examine whether there is sufficient data to make a definitive conclusion or whether recruitment should continue. The decision to continue cohort recruitment depends on the number of Clinical Benefit (CB) (defined as CR, PR or SD at week 16) in a cohort. To avoid selecting cohorts to merge based on responses, number of patients with CB are omitted from the monthly meetings.
3. Lastly, a third decision is made when patient recruitment is stopped due to a cohort having accrued enough patients. Here analysis includes all patients level data to evaluate the shared endpoints of the trials of which a cohort is completed (Table 1).

During the monthly meetings, cohorts are evaluated by the PIs from the trials that treated the patients. Decisions on expanding or closing cohorts are recorded and shared to all the participating trials through the distribution of minutes and the monthly meetings.

### Guidelines for merging cohorts

Even with cohorts that are aligned and standardised, there will still exist aggregated cohorts with limited sample sizes that may benefit from additional merging. The decision to merge these small cohorts is made by the Data-Sharing and Aggregation Working group. Merging these small cohorts together depends on whether a treatment is, for example, evaluated as tumour agnostic, such that all cohorts with a specific treatment and different tumour types may be merged. Another example is to merge mutated and amplified onco-genes if research shows that a treatment may benefit in both cases. The merging of these cohorts will be published along with the biological rationale for doing so.

## Results

Data sharing and merging in the PRIME-ROSE project have been actively ongoing since Q2 2024. As of Summer 2025, we have monitored more than 300 merged cohorts across more than 20 treatments, including more than 1,000 patients enrolled in all active trials. Twenty cohorts have progressed after interim analysis and none of the merged cohorts have been closed due to lack of clinical benefit. This means that for each cohort in which the inclusion rate increases, none of the cohorts fail to experience clinical benefit. Currently, four cohorts have completed recruitment and are under analysis. These results show that data sharing in PCM within the PRIME-ROSE project can be successfully achieved and provides a foundation for future collaborative data sharing. Moreover, the use of OMOP CDM will be determinant to demonstrate that these processes can be achieved in a harmonised and standardised manner.

## Discussion and conclusion

Sharing data among the DLCTs allows for more rapid assessment of efficacy, particularly for rare combinations of tumour type and biomarkers. The PRIME-ROSE consortium creates benefits for various stakeholders, patients, clinicians and healthcare payers by enabling decisions to be made in cohorts, which would not be possible if each cohort is considered in isolation. The data sharing structure also makes sure that the data quality is validated, as the data steward from each participating trial collects and verifies complete patient records before anonymisation and exporting it to the TSD. Additionally, the data steward in the TSD revalidates all collected records afterwards. Furthermore, using the OMOP CDM, the future of PCM is both federated and united under one umbrella. Maintaining transparency and thorough documentation in data merging is vital for the validity of the results.

Nevertheless, merging data across PCM trials is challenging. Protocols must be sufficiently aligned, as differences in inclusion and exclusion criteria, endpoints and schedule of activities can create heterogeneity that compromises comparability. Therefore, if protocol diverge to substantially, meaningful analyses may not be possible. Another practical limitation is that eCRF data from participating trials currently require manual merging by the data steward of PRIME-ROSE. A process that the OMOP CDM will streamline in the future.

Based on experiences from the PRIME-ROSE consortium, data sharing in PCM is mostly a practical endeavour with respect to communication. Aligning study design, endpoints and study procedures reduces complexity, while the monthly meetings provide a helpful forum for cohort merging and monitoring the inclusion rate of the many cohorts that exists in PRIME-ROSE. The PRIME-ROSE data sharing platform serves as a pioneering example of data sharing in practice, with the potential expansions to other PCM trials.

## Data Availability

Documents detailing the data merging process discussed in this report may be shared. However, patient information will not be shared.
